# Verification of the Dose Reduction Effect via Diluted Injection in Dual-Energy Computed Tomography Using a Human Blood Flow Phantom

**DOI:** 10.1155/2019/3512126

**Published:** 2019-04-01

**Authors:** Hironobu Tomita, Koichi Shibata

**Affiliations:** ^1^Graduate School of Healthcare Science, Suzuka University of Medical Science, 1001-1, Kishioka-cho, Suzuka, Mie 510-0293, Japan; ^2^Department of Radiology, Saiseikai Kawaguchi General Hospital, 5-11-5, Nishikawaguchi, Kawaguchi, Saitama 332-0021, Japan

## Abstract

**Purpose:**

We sought to examine the possibility of reducing the contrast medium dosage in dual-energy imaging using a saline-mixed injection with a virtual monochromatic energy method of dual-source computed tomography (CT).

**Methods:**

An X-ray CT (SOMATOM Definition Flash: Siemens, Nurnberg, Germany) was employed. The mixing ratio of contrast medium and saline was gradually changed by 10%, followed by a mixed injection into a dynamic blood flow phantom (Nemoto Kyorindo, Japan) which is a hemodynamic simulation phantom to obtain time-enhancement curves (TECs). Exactly 64 TECs were prepared for each mixing ratio by changing the energy from 40 to 75 keV for monoenergetic imaging. The relationship between the image standard deviation (SD) and the energy of the virtual monochromatic image was determined. Combinations of the mixing ratio and energy (keV), which can maintain high CT numbers and low image SDs for 3D imaging, were tested, and the reduction rate of the contrast medium was calculated.

**Results:**

The TECs for the mixed injection method changed linearly with the dilution rates. The mixing ratios were strongly correlated with the maximum CT number of the TEC (R^2^ = 0.98). Contrast CT numbers and image SDs increased by approximately 20% and 25%, respectively, as the energy decreased by 5 keV. The optimal conditions for reducing the contrast medium dose were a mixing ratio of 6:4 and 55 keV of energy.

**Conclusion:**

The virtual monochromatic energy method reduced the contrast medium dosage by up to 40% for three-dimensional CT-angio (3DCTA) tests.

## 1. Introduction

Computer tomography (CT) technology has recently made marked advances, providing various imaging methods. Currently, three-dimensional CT-angio (3DCTA) is widely used in clinical settings. In clinical contrast CT tests, a reduced contrast medium dose is beneficial for patients with renal impairment (particularly for the elderly or repeated tests) because dosage is a risk factor for the development of contrast-induced nephropathy (CIN), and its reduction minimizes the risk of CIN development [1]. Not all patients who undergo contrast CT tests have normal renal function. In Japan, approximately 13.3 million people (one-eighth of all Japanese adults) suffer from chronic kidney disease (CKD). Renal impairment can become end-stage renal failure if it is left untreated. Contrast CT testing is an effective imaging modality for patients with pulmonary infarction who must be treated rapidly. In the PIOPED II study [2], however, a contrast CT test was not indicated for 18.5% of patients suspected of having a pulmonary embolism due to high serum creatinine levels. Thus, renal impairment is common in clinical practice. However, CT tests using a reduced dose of contrast medium are useful in clinical settings because their application range is increased, and the risk of CIN development after the test is likely to decrease. A reduced contrast medium dose for CT tests is beneficial for patients.

3DCTA generates a high-intensity contrast in blood vessels, with differences in CT numbers of several hundred HU. Thus, the effects of image quality (noise) do not need to be considered in 3DCTA tests, unlike with the imaging of solid organs such as the liver. Therefore, the contrast medium dose can be markedly reduced because dual energy can maintain high CT numbers.

In previous studies, imaging with low contrast medium doses was conducted by enhancing the contrast medium sensitivity with low-tube-voltage imaging  [3, 4]. However, not all patients can undergo low-tube-voltage imaging because of the tube capacity of the device in use and the shortage of mA that occurs with larger patients. In a recent study, the contrast medium dose was reduced by approximately 30% via dual-energy imaging in clinical cases [5]. However, stable CT numbers cannot always be obtained because of differences in physical constitution, difficulties in maintaining the upper extremity venous route, and reductions in cardiac function. When the contrast medium was adjusted based on the patient's body weight, the desired CT numbers were obtained from a time-enhancement curve (TEC) by optimizing the injection rate and volume of the contrast medium [6]. However, it is difficult to adjust these factors for each test. Various methods to reduce the contrast medium dose have been examined in clinical cases to obtain the most basic rationale; however, blood flow dynamics and injection methods via phantom simulation have not been investigated. Given the aforementioned situation, we applied dual-energy CT, which enhances contrast media sensitivity, to reduce the contrast media dose in a clinical setting.

The injection volume of contrast media can be reduced in two ways: one is to use contrast media at low concentrations; the other is to lower the contrast injection rate to reduce the total amount of iodine. However, only a few formulations are commercially available at low concentrations, precluding modified concentrations. When injection rates are changed, the TEC is also changed during imaging, demanding a revision of the entire imaging protocol. In the present study, a diluted contrast medium was developed using a mixture of saline and contrast medium, enabling injection at arbitrary concentrations. In addition, images with stable contrast medium concentrations were obtained by adjusting energy (keV) after imaging. The purpose of the present study was to reduce the contrast medium dose using a mixed injection method. However, no research has demonstrated the validity of dual-energy imaging. In the present physical study, a reduction in the contrast medium dose was examined using a human blood flow phantom in actual clinical settings [6].

## 2. Materials and Methods

### 2.1. Verification System

**Figure 1:**
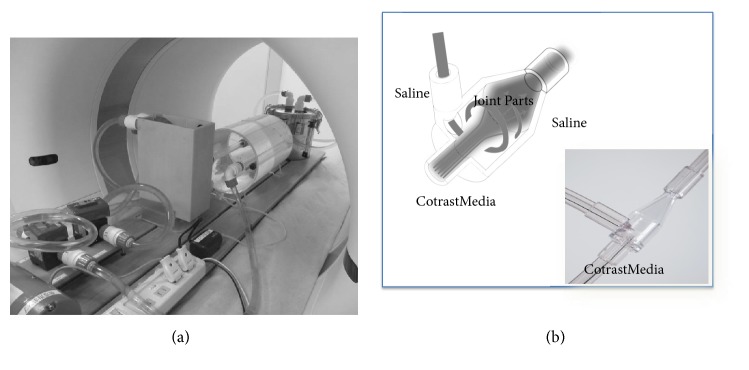
Verification system. (a) Human blood flow phantom and X-ray CT; (b) spiral flow tube.

The human blood flow phantom (Nemoto Co. Ltd, Tokyo, Japan), which can reproduce TECs in human arteries as reported in a previous paper [7], was used to measure TECs (Figure 1). The human blood flow phantom was established by assuming a 60-kg body weight, 5-L circulating water, and an intra-arterial CT angiography. The contrast medium injection rate was 3.5 mL/sec, and 42 mL of Isohexol contrast medium (350 mgI/mL) was administered. An acrylic cylinder with a diameter of 200 mm in a phantom imaging unit was filled with water. Acrylic tubes, simulating arteries and veins, were placed on both sides of the cylinder to simulate arterial and venous blood flows, through which the contrast medium was circulated.

An X-ray CT (SOMATOM Definition Flash: Siemens, Nurnberg, Germany) and spiral tube (Spiral Flow Tube DS-017: Nemoto Co. Ltd, Tokyo, Japan) [8] were used for the dilution of contrast media and saline (Figure 1).

### 2.2. Verification Procedure

#### 2.2.1. Comparison between DIC and VMI

Dual imaging on CT can be achieved using two methods. One is the dual-energy imaging composition (DIC) method that generates images by mixing two images of different tube voltages at an arbitrary ratio. The other is the virtual monoenergetic imaging (VMI) method that generates a monoenergetic image of arbitrary energy by calculating a virtual monoenergetic image from two images of different tube voltages. To determine which imaging technique is more advantageous for contrast media reduction, we investigated the imaging characteristics of the contrast media using both DIC and VMI.

For this investigation, we performed dual-energy imaging using the human blood flow dynamic phantom. The tube voltages were 100 kV and 140 kV with a Sn filter. To adjust image quality and image standard deviation (SD), we set the image SD to 11 under 100 kV and the mAs to 180 mAs (CTDI_VOL_ of 12 mGy).

**Figure 2:**
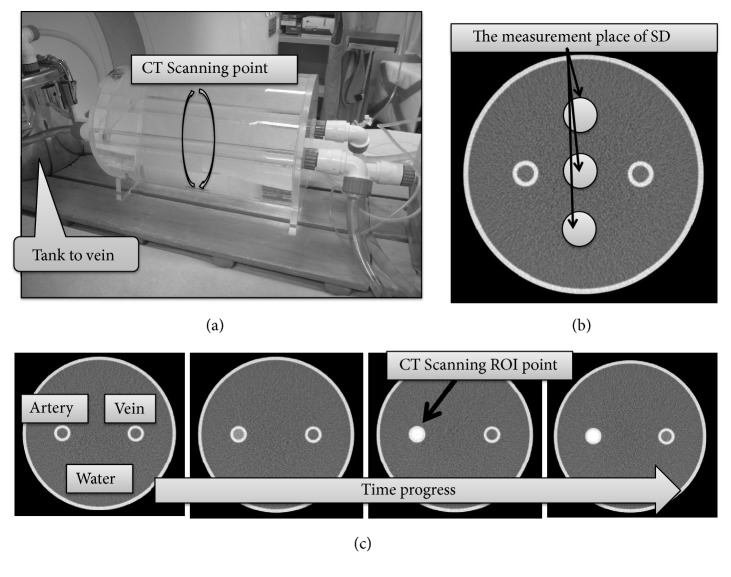
Blood circulation mock phantom. (a) CT scanning point; (b) the measurement location of the image SD; (c) CT scanning region of interest (ROI).

(1) The contrast media Isohexol, at 350 mg/mL of the total 42 mL volume, was injected into the human blood flow phantom at rate of 3.5 mL/s. The total injection time was 12 s. The ROI was set at the arterial region of the phantom (see Figure 2), and the images were acquired temporarily. Image acquisitions were performed every 3.25 s after the contrast media injection for 12 acquisitions. The total acquisition time was 39 s.

(2) We reconstructed the dual-energy images using the DIC and VMI methods and created graphs showing the temporary TEC changes at each imaging energy. The longitudinal axis of the graphs represents time, and the vertical axis represents the CT number. Using these graphs, we investigated the difference in the TECs at 100 kVp, which is used in conventional CT angiography, and at the other imaging energies mentioned below. For example, M0.2 indicates a mixed image comprising 20% of a 100-kVp image and 80% of a 100-kVp image.

  Acquired images: 100 kVp, 140 kVp,
  DIC images: M0.2 (100 kVp 20% 140 kVp 80%), M0.5 (100 kVp 50% 140 kVp 50%) M0.7 (100 kVp 70% 140 kVp 30%), M0.9 (100 kVp 90% 140 kVp 10%),
  VMI images: 40 keV 45 keV 50 keV 55 keV 60 keV 65 keV.

#### 2.2.2. Change in Energy via VMI and CT Number

We created graphs to show the changes in the CT number of the contrast media, water, and fat (from a pig) for every 5 keV from 40 to 190 keV using VMI. The longitudinal axis of the graphs shows the virtual monoenergetic energy, and the vertical axis shows the CT numbers. We used 300 HU, 200 HU, and 100 HU of diluted contrast media at 12 kVp. The acquisition conditions for the CT were 100 kV and 140 kV (using a Sn filter).

#### 2.2.3. The Optimization of the Monoenergetic Imaging Conditions

We investigated the optimum dilution rate, monoenergetic energy, and CT number for the contrast media reduction. The experimental procedures are described below.

Contrast medium: the saline ratio was changed by 10% from 2:8 to 10:0, and a mixture of contrast medium and physiological saline was injected into the human blood flow phantom. The arterial portion of the phantom (Figure 2) was imaged to record the time course of the CT numbers. The imaging conditions were 100 kV and 140 kVp (using an Sn filter). Image quality and SD were adjusted at 180 mAs (CTDI_VOL_ of 12 mGy) to achieve an SD of 11 with a 1-mm slice thickness at 100 kV. Images were obtained every 3.25 s (12 times for 39 s) after the start of contrast medium injection. The CT numbers were plotted over time to create a TEC. For the TEC data from 2:8 to 10:0, the energy was changed by 5 keV from 40 to 75 keV to create VMI images. Thus, 64 TECs were prepared under the various mixture injection conditions. We determined the relationship between the temporal changes of the CT numbers and the monoenergetic energy of VMI by created TEC graphs in which the longitudinal axis represents time, and the vertical axis represents the CT numbers.

#### 2.2.4. The Relationship between Maximum CT Numbers and Mixing Rate

We investigated the relationship between the maximum number of TECs at each monoenergetic energy and the mixing ratio of contrast media and saline. The maximum CT numbers were obtained from the TECs ranging from 40 to 75 keV, as indicated on the graphs in which the longitudinal axis shows the monoenergetic energy, and the vertical axis shows the CT numbers.

#### 2.2.5. SD Characteristics of Monoenergetic Image

Mean SDs at three water positions in the blood flow phantom (Figure 2) were determined for each monoenergetic energy.

#### 2.2.6. Determination of Optimal Imaging Conditions

A tube voltage of 100 kVp, which is routinely applied for imaging at our hospital, was used as a reference. Combinations that were able to maintain a CT number of > 300 HU and an image SD of 15 or below (a TH of 1 mm and a reconstruction function of D30) were determined to calculate the contrast medium reduction rates [1]. These numbers are the minimum requirements needed to prepare 3DCTA.

## 3. Results and Discussion

### 3.1. Comparison between DIC and VMI

**Figure 3:**
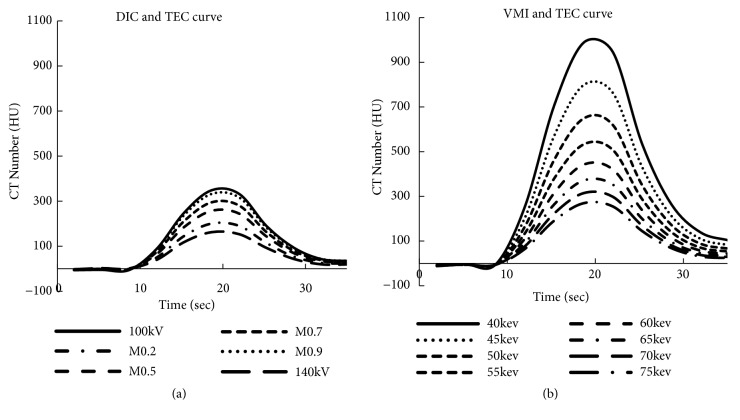
The DIC and VMI of the contrast media sensitivity. (a) The TECs of the DIC images, which show the differences among the mixing ratios of 100 kVp and 140 kVp. (b) The TECs of the VMI images, which show the differences among the monoenergetic energies. Injection condition: saline : contrast media, 0 : 10.

Figure 3(a) shows the TECs of the DIC images, revealing the differences among the mixing ratios of the 100 kVp and 140 kVp images.  Figure 3(b) shows the TECs of the VMI images and the differences among the monoenergetic energies. The 100-kVp curve had the maximum CT number for DIC, and (compared with DIC) VMI showed a higher increase in the CT number. VMI was superior to DIC under 65 keV, exceeding the maximum number of 300 HU of DIC.

### 3.2. Change in the Energy Regarding VMI and CT Number

**Figure 4:**
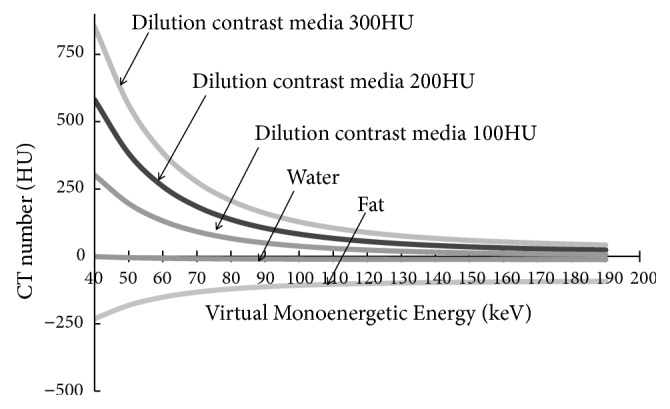
Changes in the energy with regard to VMI and CT number.

The changes in the contrast media, water, and fat (from a pig) with regard to the monoenergetic energy in VMI were more obvious when the density of the contrast media was higher or the monoenergetic energy was lower. For example, the CT number of the contrast media, which was diluted to 300 HU, more than doubled to 851 HU at 40 keV. On the other hand, a small change in the CT number was observed for water, and the CT number decreased for fat, which might have opposing characteristics to those of the contrast media. (Figure 4)

### 3.3. The Optimization of the Monoenergetic Imaging Conditions

**Figure 5:**
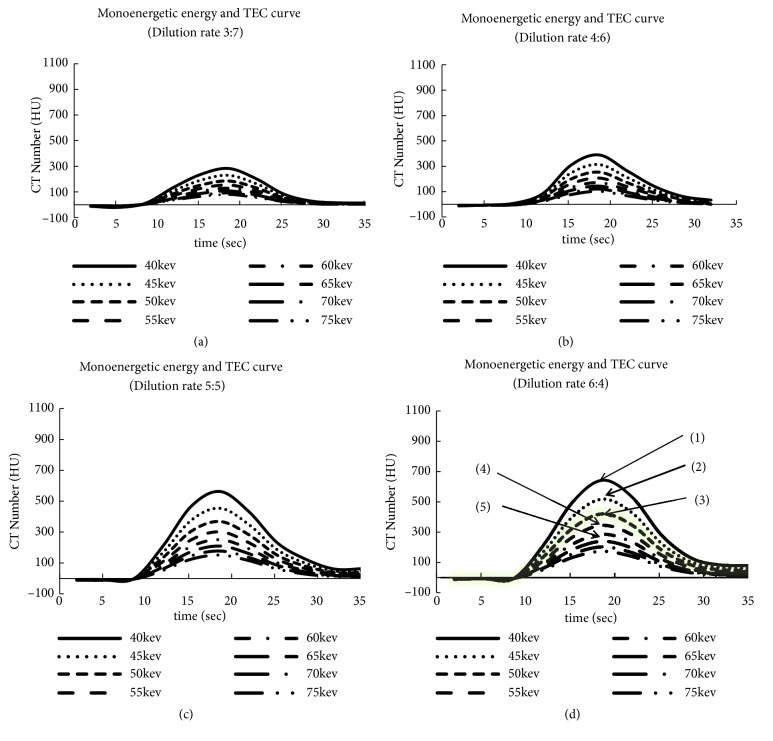
Monoenergetic energy and TEC. (a) Dilution rate 3:7; (b) dilution rate 4:6; (c) dilution rate 5:5; (d) dilution rate 6:4.

**Figure 6:**
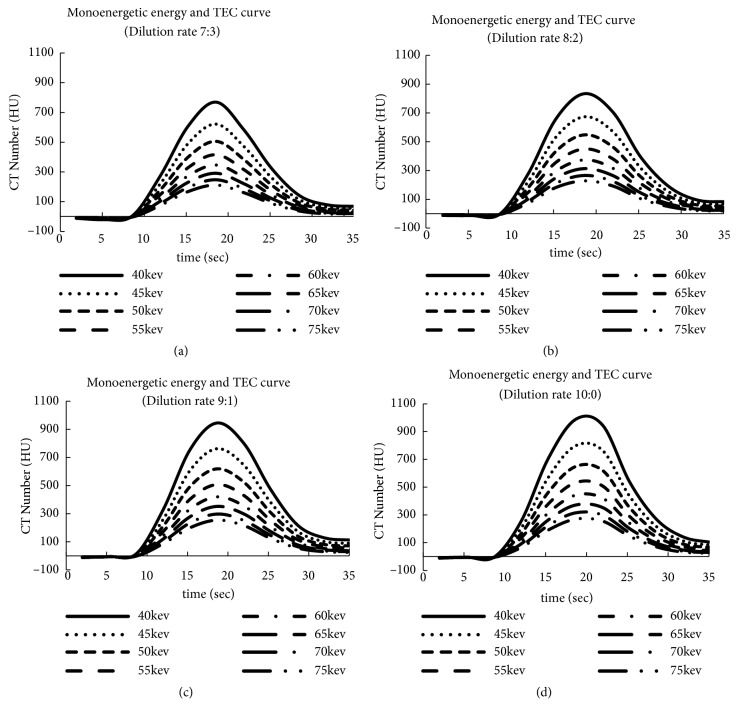
Monoenergetic energy and TEC. (a) Dilution rate 7:3; (b) dilution rate 8:2; (c) dilution rate 9:1; (d) dilution rate 10:0.

Figures 5 and 6 show the TEC results obtained using VMI by changing the dilution rate as well as the relationships among dilution rate, monoenergetic energy, and CT number. The vertical and horizontal axes show the CT number and time (s), respectively, and each TEC ranges from 40 to 75 keV monoenergetic energy. The difference between Figures 5(a)–5(d) and Figures 6(a)–6(d) is the dilution rate. For example, the TECs started to increase approximately 10 s after the start of the injection, reached a peak at approximately 18 s, and gradually decreased by 30 s for a dilution rate of 6:4 (Figure 5(d)). The TEC at 40 keV had the highest peak compared with the other TECs, and its maximum CT number was 642 HU (Figure 5(d){1}). When the monoenergetic energy increased, the maximum CT numbers decreased (e.g., 518 HU at 45 keV [2], 345 HU at 50 keV [3], and 285 HU at 55 keV [4]). The same tendency was observed in the other figures (i.e., Figures 5(a)–5(c) and Figures 6(a) and 6(b)). The maximum CT numbers increased linearly by approximately 20%, following a decrease of 5 keV for the monoenergetic energy at any dilution rate using VMI.

**Figure 7:**
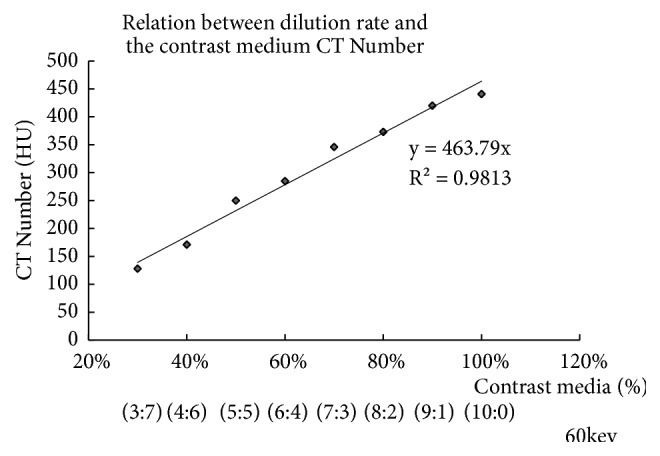
The dilution rate of contrast media and saline, exhibiting a strong correlation (R^2^ = 0.98).

Figure 7 shows the relationship between the dilution rate and the maximum CT number of the TECs of Figures 5 and 6 at 60 keV of monoenergetic energy. The shapes of the TECs prepared using the diluted injection method changed linearly with the dilution rate. The maximum CT number was strongly correlated with the dilution rate ratio of the contrast medium and saline (R^2^ = 0.98), demonstrating a linear rise in the CT numbers with the increase in the dilution rate.

### 3.4. The Relationship between Maximum CT Number and Dilution Rate

**Figure 8:**
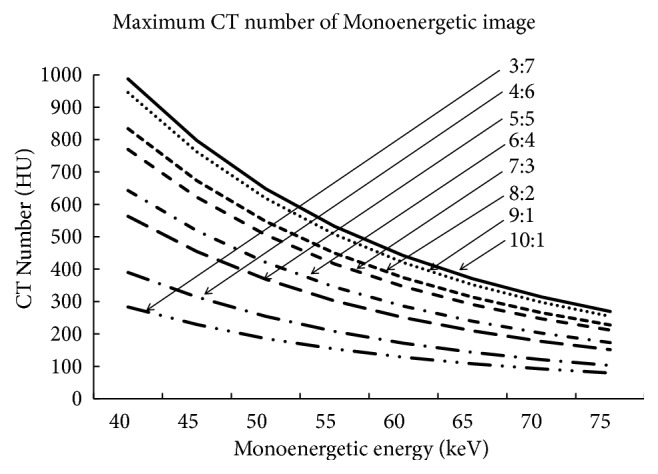
The maximum CT number per dilution rate. The maximum CT number per each keV resulted in a higher number if the diluted ratio was higher. If keV was lower, the maximum CT number was higher. Finally, for 100% contrast (0% saline) at 40 kV, the CT number reached 1000 HU.

Figure 8 shows the change in the maximum CT number per each dilution rate following the change in monoenergetic energy. The maximum CT numbers for the TEC at each energy increased when the dilution rate of the contrast medium or virtual energy decreased, reaching 1,000 HU at 10:1 and 40 keV.

### 3.5. SD Characteristics of Monoenergetic Images

**Figure 9:**
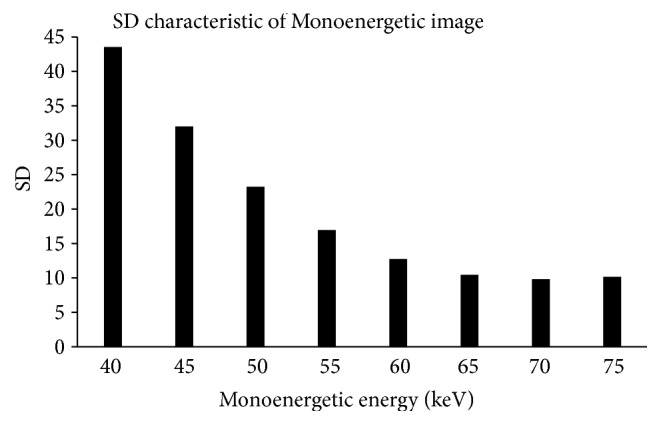
The SD characteristic of a monoenergetic image. Image SD increased by 25% for each reduction of 5 kV between 40 and 65 kV; it did not increase and maintained a consistent level for energies higher than 65 kV.

Figure 9 shows the results of the change in image SD with regard to the change in monoenergetic energy. Image SD increased by approximately 25% for every 5-keV decrease between 40 and 65 keV and remained approximately constant above 65 keV. We confirmed that image SD changes with monoenergetic energy, and an increase in image SD of 40 - 50 keV was clearly realized with relatively higher CT numbers.

### 3.6. Determination of Optimal Imaging Conditions

**Figure 10:**
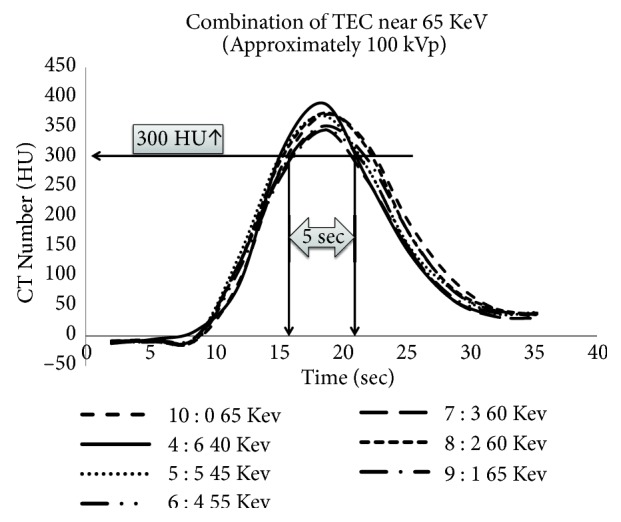
Determination of the best combination of dilution rate and monoenergetic energy using VMI. The combinations that maintained enhancement above 300 HU were 10:0 at 65 kV, 4:6 at 40 keV, 5:5 at 45 keV, 6:4 at 55 keV, 7:3 at 60 keV, 8:2 at 60 keV, and 9:1 at 65 keV. In addition, the dilution ratio of 6:4 at 55 keV only provided an enhancement level of 300 HU for 5 s, which corroborates the TEC using single-energy scanning at 100 kV.

The combinations capable of maintaining the CT number above 300 HU for the 5-s acquisition time [9] were 10:0 and 65 keV, 4:6 and 40 keV, 5:5 and 45 keV, 6:4 and 55 keV, 7:3 and 60 keV, 8:2 and 60 keV, and 9:1 and 65 keV (Figure 10). Of these combinations, the dilution rate of 6:4 and a monoenergetic energy of 55 keV maintained the image at an SD of 15 or below (TH of 1 mm and a reconstruction function of D30).

We confirmed that VMI is able to obtain higher CT numbers than DIC by adjusting the monoenergetic energy (see Figure 3). This result indicates that VMI is a more effective method for reducing the contrast media dose.

**Figure 11:**
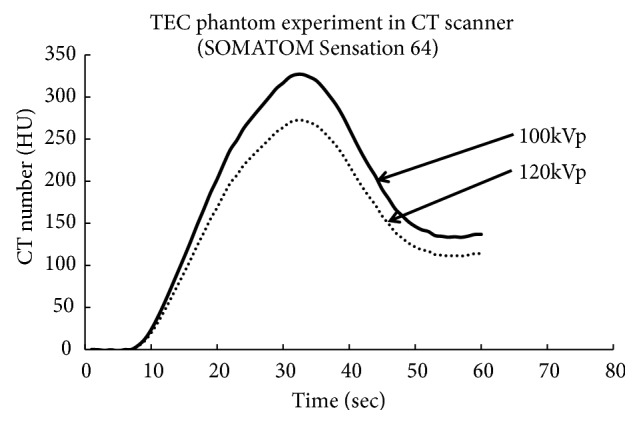
TECs of 120 kV and 100 kV obtained from conventional CT images with low kVp imaging. At an injection rate of 3.0 mL/s, 75 mL (60 kg) was injected for 25 s, and the amount of the circulation was set by assumption. Under low kVp imaging, lower kVp (100 kVp) provides higher CT numbers for the contrast material.

Figure 11 shows the experimental results that demonstrate the increase in CT number acquired from the conventional CT image with low kVp imaging. This TEC was created by injecting the total 75-mL volume of contrast media at 3.0 mL/s (for 25 s) into the human blood flow phantom to simulate a human body of 60 kg at 100 and 120 kVp. Low kVp imaging at 100 kVp can obtain relatively higher CT numbers but does not achieve the increases in CT number obtained using the VMI method.

As Table 1 shows, the tube voltage and sensitivity ratio of the contrast medium can be easily measured. Thus, a mixed injection based on sensitivity ratios should facilitate imaging. CT numbers were measured from the images acquired under various kVp with diluted contrast media of 5-20 mgI/mL via conventional scan. The sensitivity was calculated by normalizing each CT number with the CT number at 120 kVp (100% at 120 kVp).

The present study simulated the examination of vessels, which have relatively high image contrast on dual-energy CT. For examinations with normal image contrast, Albrecht et al. reported the advantages of the VMI method based on a study that compared monoenergetic images at 55 and 70 keV and DIC image of M0.3 (80 kVp 30% and 140 kVp 70%) obtained from 44 patients who were diagnosed with head and neck squamous cell carcinoma.

Hardie AD1 reported that a monoenergetic image at 55 keV showed better results for image noise reduction and diagnostic capability using the VMI method compared with a conventional CT image at 120 kVp. That result was obtained in an angiographic study of pancreas cancer (12 cases of adenocarcinoma, 5 cases of neuroendocrine, and 7 cases of cystic tumors). Although Hardie did not test a contrast media reduction, we also obtained similar results in which 55 keV was the best monoenergetic energy for maintaining less image noise and an appropriate CT number. We confirmed that the VMI method can be applied to diagnose cancer and image other organs that have a rich blood flow.

Depending on the CT numbers of the original images, 1,000 HU or above can be achieved using 40 keV for a virtual monochromatic image. However, this setting is impractical because an image SD of 40 or above is obtained in clinical settings, which is approximately four times greater than single-energy imaging at 100 kVp. Given the commonly used dosage range, the limit is approximately 55 keV. In addition, the combination of 6:4 and 55 keV (see Figure 11) might be suitable for maintaining 300 HU or above for approximately 5 s (imaging time) and obtaining the same 3D image quality (an SD of 15 or less) for the TEC at 100 kV during single-energy imaging (Figure 1).

The present study, performed using a SOMATOM Definition Flash system (Siemens), found that the combination of 6:4 and 55 keV was the optimum condition for VMI. However, the relationship between keV and image SD might differ with imaging devices, limiting this study to the present system. With the development of new devices, the contrast medium dosage might be further reduced.

The contrast medium dose can be reduced in dual-energy imaging, and any CT number can be obtained by adjusting the energy to modify the contrast CT numbers, thereby generating stable 3DCT images. Reliability is critical for CT tests. For example, the same images can be generated even with a fixed threshold number of volume rendering during the preparation of 3DCTA images, as long as the blood CT numbers are constant.

The diluted injection of saline and contrast medium enables energy adjustment for each test case by changing the mixing ratios with the same contrast and imaging timing, and it is easily introduced to clinical settings. Using this method, highly reliable 3DCTA images can be acquired, and the contrast medium dosage reduction rate can be calculated.

In the present study, the diluted injection of contrast medium was highly reliable in clinical settings because the CT numbers of the contrast media changed linearly with the dilution rate as expected.

Currently, a 180- to 300-mg range of iodine is administered as a contrast medium (24-50 mL of 300 mgI/mL) to visualize the arteries on CT angiography [7]. Based on the results of the present study, this dose can be reduced by approximately 30% for imaging in our hospital.

In recent years, there are various types of devices, such as dual source, fast kV switching, dual layer detector, and split-filter types, that can be used for clinical dual energy imaging. Jacobsen [10] et al. have reported variations in VMI sensitivity, as a result of experiments, between these different device types. Washio [11] et al. also compared VMI sensitivity of fast kV switching to that of dual layer detector. But they found only slight differences (less than 10%).

Here, we studied 3DCTA. Since it has a high contrast region of 300 HU or more, which is within the acceptable range of error, we consider that research can be diverted towards sensitivity and infusion dilution ratio of the contrast agent in all methods.

However, since changes in VMI image quality (such as SD) due to energy changes depend on CT (dual source, high-speed kV switching, dual layer detector, and split filter type), we assume that some consideration such as dose is required.

In addition, X-ray CT devices capable of multi-kVp settings will be widely used in clinical practice. The diluted injection method, combined with low-tube-voltage imaging, will reduce both radiation and contrast medium doses in the clinical field.

## 4. Conclusions

The present phantom experiment demonstrated that vascular CT numbers changed with the mixing ratios and generated stable TECs using the diluted injection method. In addition, the contrast medium dosage was reduced up to approximately 40% in 3DCTA tests using the VMI method of dual-energy CT. This method can be easily applied to clinical practice using mixed angiography and dual-energy CT without changing the imaging and acquisition timing. Furthermore, contrast CT numbers can be finely adjusted via VMI, providing stable 3D images.

## Figures and Tables

**Table 1:** Sensitivity for contrast media under conventional CT. CT numbers were measured from the images acquired under various kVp with diluted contrast media of 5-20 mgI/mL via conventional scans. The sensitivity was calculated by normalizing each CT number with the CT number at 120 kVp (100% at 120 kVp).

Contrast media	Tube voltage				
	70kVp	80kVp	100kVp	120kVp	140kVp
5mgI/mL	1.9	1.6	1.2	1.0	0.9
10mgI/mL	1.9	1.6	1.2	1.0	0.9
15mgI/mL	2.0	1.6	1.2	1.0	0.9
20mgI/mL	2.0	1.6	1.2	1.0	0.8

SIEMENS SOMATOM Definition Flash

## Data Availability

The imaging and other data used to support the findings of this study are included within the article.
