# Corrigendum to “Verification of the Dose Reduction Effect via Diluted Injection in Dual-Energy Computed Tomography Using a Human Blood Flow Phantom”

**DOI:** 10.1155/2019/5109419

**Published:** 2019-06-19

**Authors:** Hironobu Tomita, Koichi Shibata

**Affiliations:** ^1^Graduate School of Health Science, Suzuka University of Medical Science, 1001-1, Kishiokacho, Suzuka, Mie 510-0293, Japan; ^2^Department of Radiology, Saiseikai Kawaguchi General Hospital, 5-11-5, Nishikawaguchi, Kawaguchi, Saitama 332-0021, Japan

In the article titled “Verification of the Dose Reduction Effect via Diluted Injection in Dual-Energy Computed Tomography Using a Human Blood Flow Phantom” [1], the first affiliation was written incorrectly. The corrected authors' list and affiliations are shown above.
